# Supplementary figures and images for: Mucosal CD8+ T cell responses induced by an MCMV based vaccine vector confer protection against influenza challenge
Source: PLoS Pathog. 2019 Sep 16;15(9):e1008036. doi: 10.1371/journal.ppat.1008036 (PMC6763260; doi:10.1371/journal.ppat.1008036)

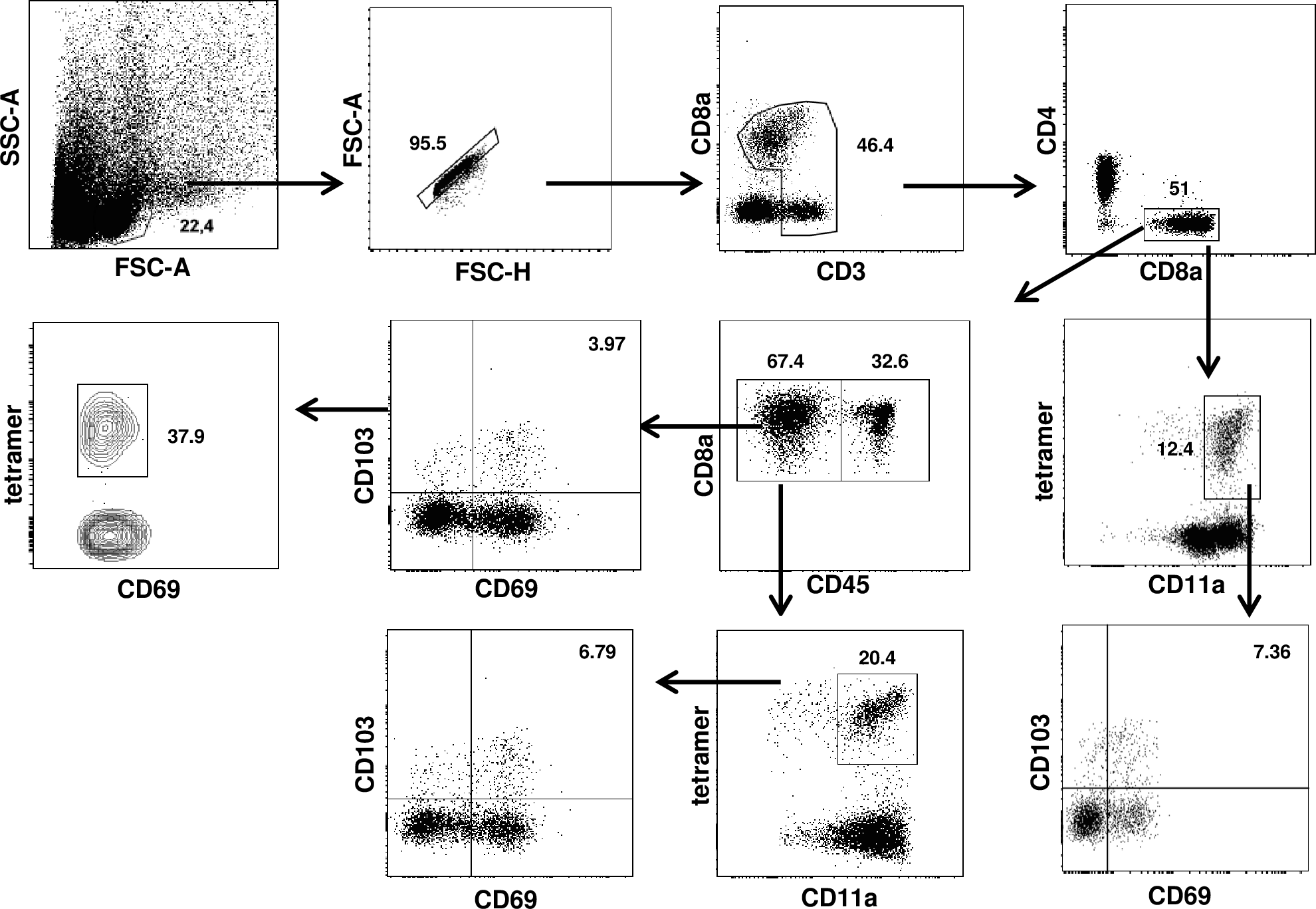

Supplement: S1 Fig — BALB/c mice were immunized with 2 x 10^5^ PFU MCMVIVL via the i.p. or i.n. route. During latency (> 3 months p.i), Leukocytes from blood, spleen and lungs were stained with cell surface markers CD3, CD4, CD8, CD11a, CD69, CD103, KLRG1, CD62L, IVL-tetramer and analyzed by flow cytometry. For in vivo labeling, anti-CD45 antibodies were injected intravenously 3–5 min before mice euthanasia. Gating strategy of each cell subset is shown. (TIF) [file ppat.1008036.s001.tif]

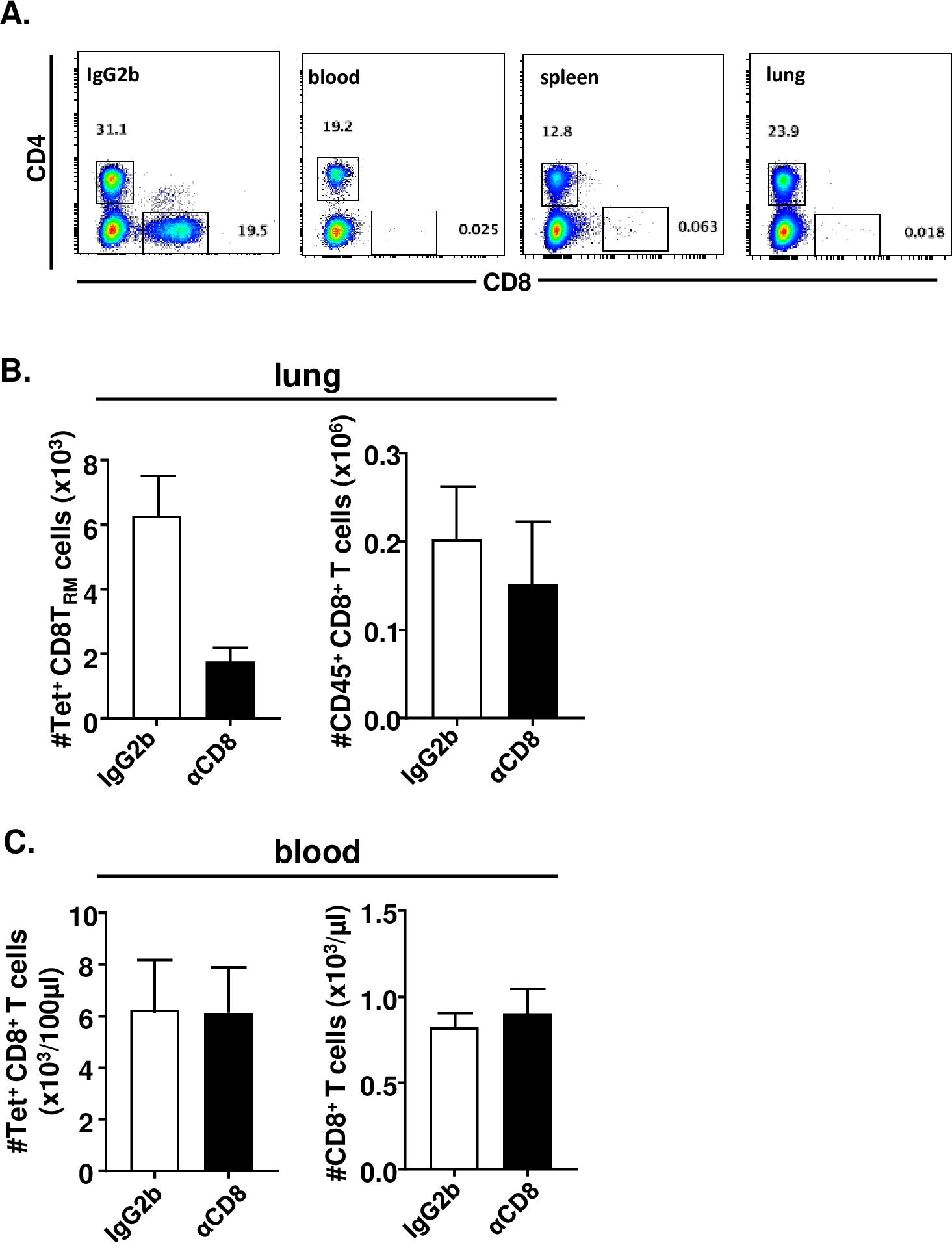

Supplement: S2 Fig — BALB/c mice were immunized with 2 x 10^5^ PFU MCMVIVL by the i.n. route. (A) During latency (> 3 months p.i), mice were injected 200 μg αCD8 antibody (i.p.) to deplete total CD8+ T cells. Same amount of IgG2b antibody was given as isotype control. Leukocytes from blood, spleen and lungs were analyzed by flow cytometry and representative flow cytometric panels in blood, spleen and lungs on day 1 post-depletion are shown. (B-C) Mice were administered with 10 μg αCD8 antibody (i.n.) to deplete airway CD8+ T cells in the lungs or IgG2b as a control. (B) The number of IVL-tetramer+ CD8TRM cells and circulating CD8+ T cells (CD45+) in the lungs on day 1 post airway CD8+ T cell depletion. (C) The number of IVL-specific and total CD8+ T cells in the peripheral blood on day 1 post airway CD8+ T cell depletion. Bars indicate means, error bars are SEM. (TIF) [file ppat.1008036.s002.tif]

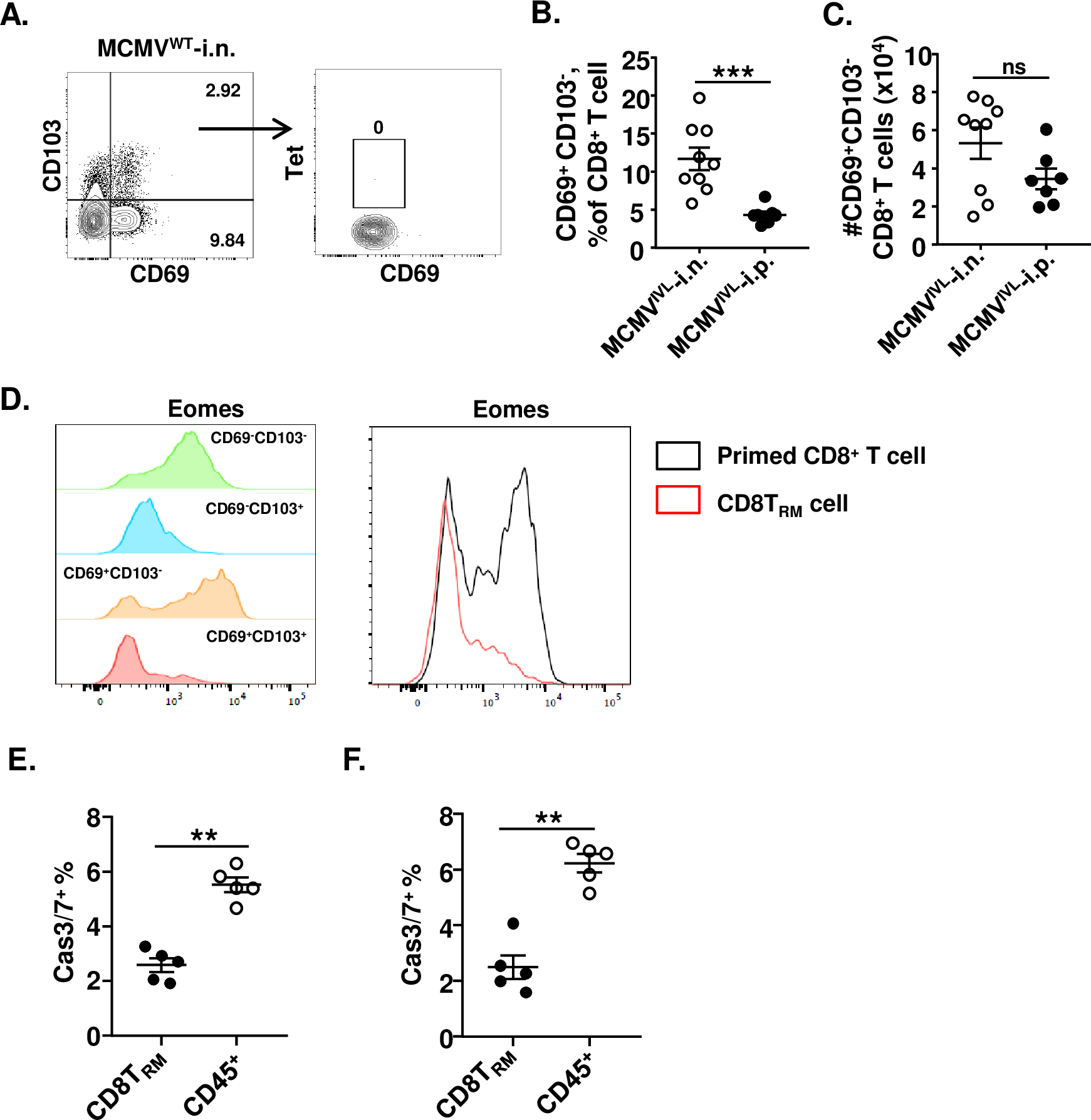

Supplement: S3 Fig — BALB/c mice were immunized with 2 x 10^5^ PFU MCMVWT via the i.n. route. During latency (> 3 months p.i), leukocytes were isolated from lungs, stained with cell surface markers against CD4, CD8, CD69, CD103 before flow cytometry. (A) Representative dot plots of CD8TRM and IVL-specific CD8TRM cells. (B, C) BALB/c mice were immunized with 2 x 10^5^ PFU MCMVIVL via the i.n. or i.p. route. (B) Percentage of CD69+CD103-CD8+ T cells in the lungs. (C) The number of CD69+CD103-CD8+ T cells in the lungs. (D) Eomes expression on different subsets of CD8+ T cells in the lungs. (E) Percentage of caspase3/7+ cells among CD8TRM and circulating CD8+ T (CD45+) cells. (F) Percentage of caspase3/7+ cells among tetramer+ CD8TRM and circulating CD8+ T (CD45+) cells. Two independent experiments were performed and pooled data are shown. Each symbol represents one mouse, n = 5–9. Group means +/- SEM are shown. Significance was assessed by Mann-Whitney U test. **P <0.01, ***P <0.001, ns: no significance. (TIF) [file ppat.1008036.s003.tif]

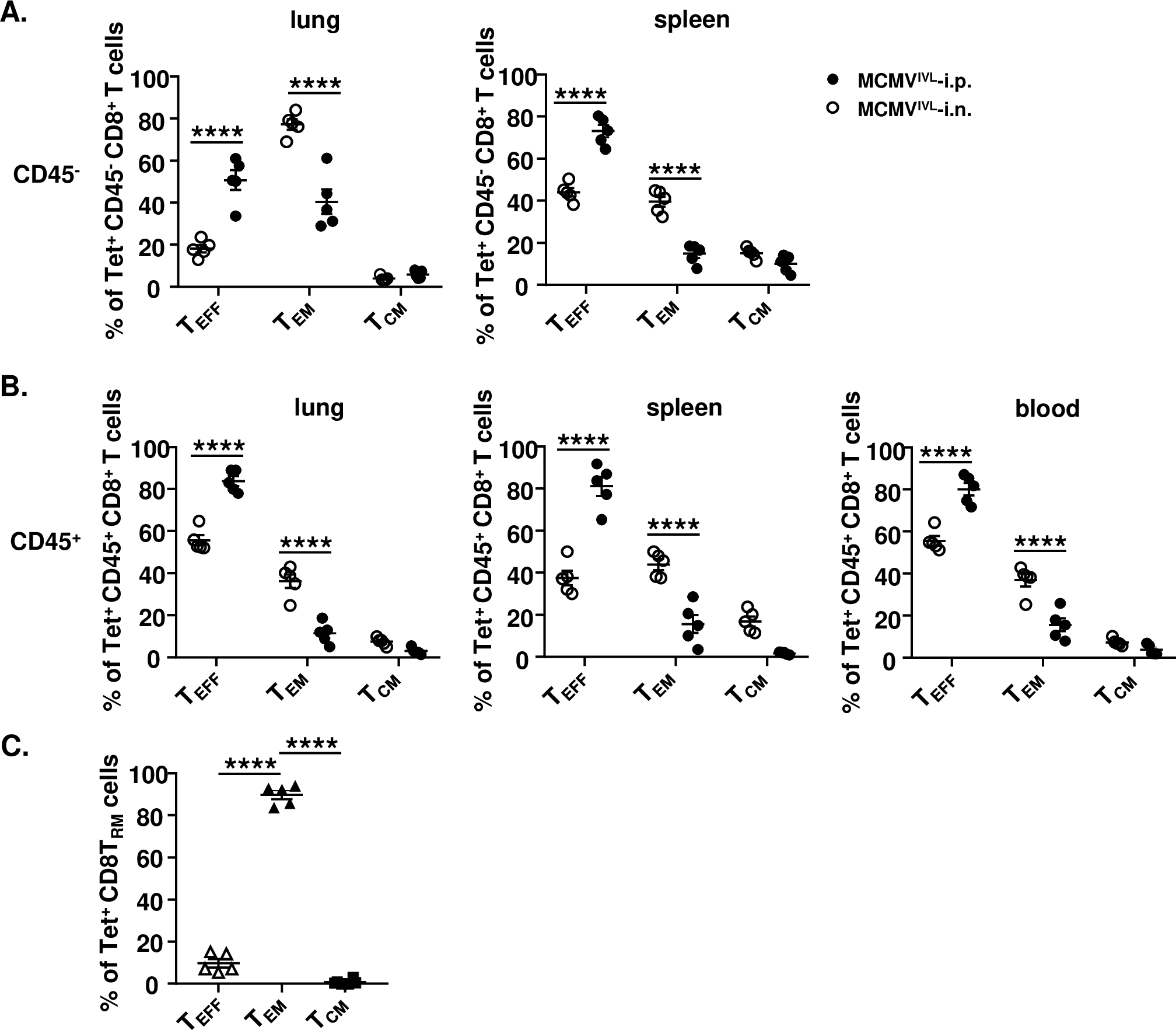

Supplement: S4 Fig — BALB/c mice were immunized with 2 x 10^5^ PFU MCMVIVL via the i.p. or i.n. route. During latency (> 3 months p.i), anti-CD45 antibodies were injected intravenously 3–5 min before mice euthanasia. Leukocytes from blood, spleen and lungs were stained with cell surface markers CD3, CD4, CD8, CD11a, KLRG1, CD62L, IVL-tetramer and analyzed by flow cytometry. TEFF cells are defined as KLRG1+CD62L-, TEM as KLRG1-CD62L-and TCM as KLRG1-CD62L+. (A) The percentages of each phenotype subset among CD45- tetramer+ CD8+ T cells in the lungs and spleen. (B) The percentages of each phenotype subset among CD45+ tetramer+ CD8+ T cells and tetramer+ CD8TRM cells in the lungs, spleen and blood. (C) The percentages of each phenotype subset among tetramer+ CD8TRM cells in the lungs. Two independent experiments were performed and pooled data are shown, n = 5. Each symbol represents one mouse. Group means +/- SEM are shown. Significance was assessed by One-way ANOVA and Two-way ANOVA test. ****P <0.0001. (TIF) [file ppat.1008036.s004.tif]

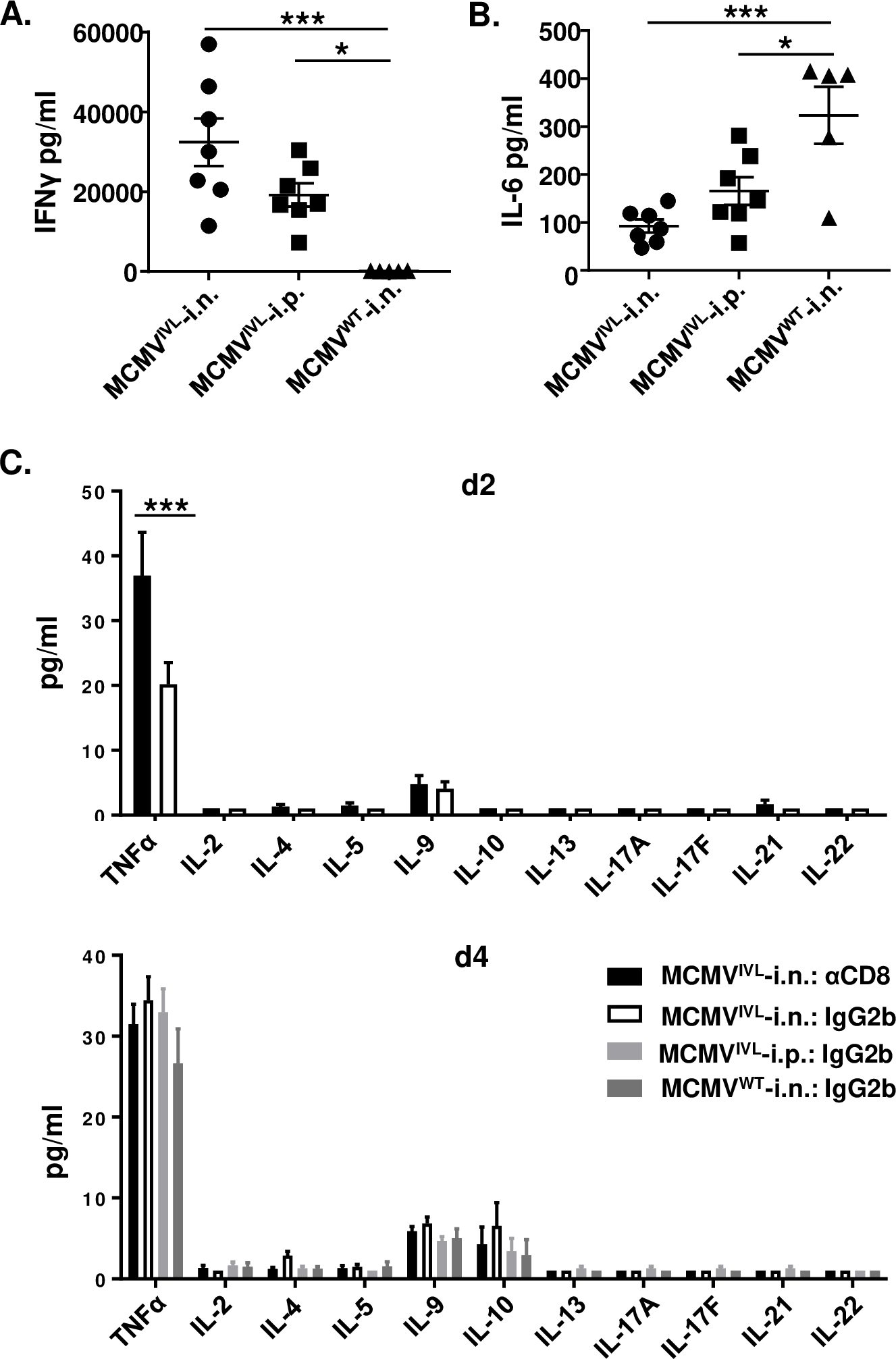

Supplement: S5 Fig — BALB/c mice were immunized with 2 x 10^5^ PFU MCMVIVL via the i.n. or i.p. route or with MCMVWT via the i.n. route. During latency (> 3 months p.i), MCMVIVL (i.n.) immunized mice were administered with 10 μg αCD8 or 10 μg IgG2b antibody (i.n.). MCMVIVL (i.p.) and MCMVWT (i.n.) immunized mice were administered with 10 μg IgG2b antibody (i.n.). One day later, animals were challenged with IAV (PR8) (i.n., 1100 FFU). On day 2 and day 4 post-challenge, BALF was harvested and measured cytokines production by bio-plexing. The concentration of (A) IFNγ and (B) IL-6 in the BALF on day 4 post-challenge. Two independent experiments were performed and pooled data are shown. Each symbol represents one mouse, n = 5–7. Group means +/- SEM are shown. (C) Cytokine concentrations in the BALF in different immunization group on day 2 and day 4 post-challenge. Bars indicate means, error bars are SEM. Two independent experiments were performed and pooled data are shown. Each symbol represents one mouse, n = 5–7. Significance was assessed by One-way ANOVA test. *P <0.05, ***P <0.001. (TIF) [file ppat.1008036.s005.tif]

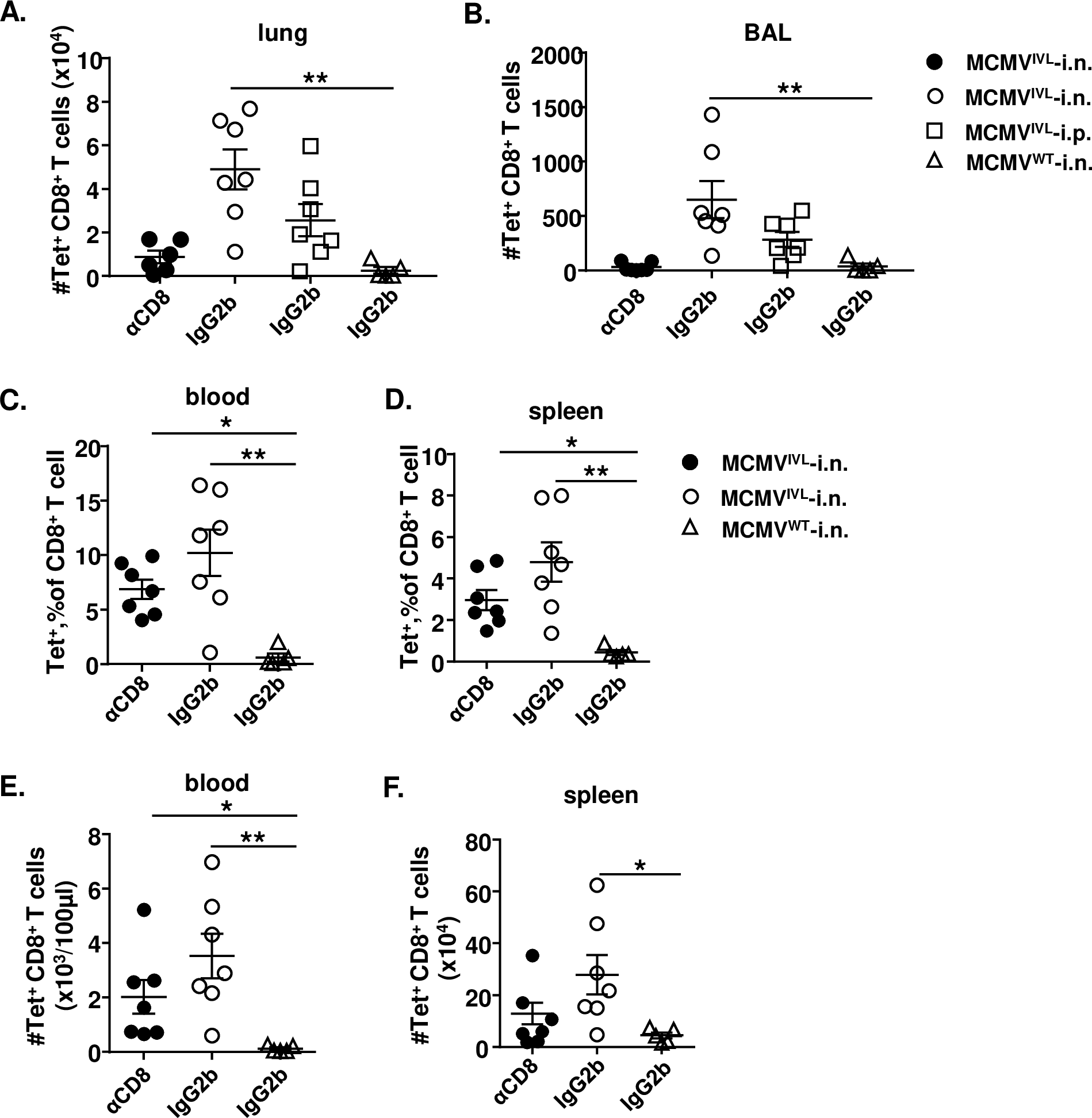

Supplement: S6 Fig — BALB/c mice were immunized with 2 x 10^5^ PFU MCMVIVL by the i.n. or i.p. route or with MCMVWT by the i.n. route. During latency (> 3 months p.i), mice were challenged with IAV (PR8) (i.n., 1100 FFU) one day after airway CD8+ T cell depletion. On day 4 post-challenge, anti-CD45 antibodies were injected intravenously 3–5 min before mice euthanasia. Leukocytes were isolated from lung, BAL, blood and spleen. (A-B) Count of IVL-specific CD8+ T cells in the lungs (A) and BAL (B). (C-D) Percentage of IVL-specific CD8+ T cells among CD8+ T cells in the blood (C) and spleen (D). (E-F) Count of IVL-specific CD8+ T cell in the blood (E) and spleen (F). Two independent experiments were performed and pooled data are shown. Each symbol represents one mouse, n = 5–7. Group means +/- SEM are shown. Significance was assessed by One-way ANOVA test. *P <0.05, **P <0.01. (TIF) [file ppat.1008036.s006.tif]

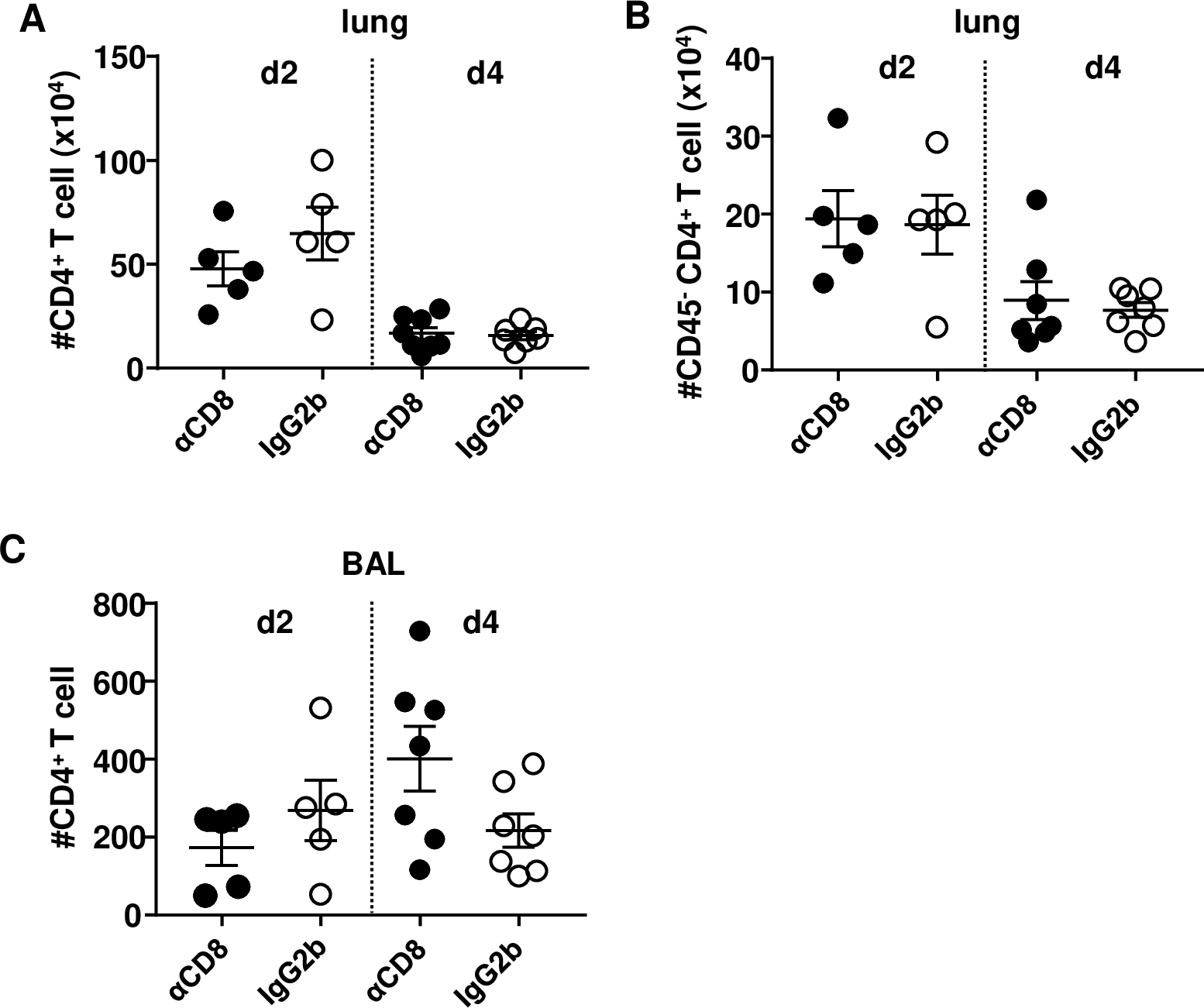

Supplement: S7 Fig — BALB/c mice were immunized with 2 x 10^5^ PFU MCMVIVL by the i.n. route. During latency (> 3 months p.i), mice were challenged with IAV (PR8) (i.n., 1100 FFU) one day after airway CD8+ T cell depletion. On day 2 and day 4 post-challenge, anti-CD45 antibodies were injected intravenously 3–5 min before mice euthanasia. Leukocytes were isolated from lungs and BAL. CD4+ T cell numbers in the lungs are shown. (A) The number of total CD4+ T cells. (B) The number of CD45- CD4+ T cells. (C) The number of CD4+ T cells in the BAL. Two independent experiments were performed and pooled data are shown. Each symbol represents one mouse, n = 5–7. Group means +/- SEM are shown. (TIF) [file ppat.1008036.s007.tif]
